# Characterization of ultrasonically extracted flaxseed polysaccharide gum and assessing its lipid‐lowering potential in a rat model

**DOI:** 10.1002/fsn3.3045

**Published:** 2022-10-17

**Authors:** Muhammad Nadeem Akhtar, Anees Ahmed Khalil, Ahmed Bilal, Muhammad Afzaal, Tabussam Tufail, Rabia Saeed, Rabia Siddique, Arash Nemat, Muhammad Faisal Manzoor

**Affiliations:** ^1^ University Institute of Diet and Nutritional Sciences, Faculty of Allied Health Sciences The University of Lahore Lahore Pakistan; ^2^ Department of Food Science, Faculty of Life Sciences Government College University Faisalabad Faisalabad Pakistan; ^3^ Department of Microbiology University of Health Sciences Lahore Lahore Pakistan; ^4^ Department of Chemistry Government College University Faisalabad Faisalabad Pakistan; ^5^ Department of Microbiology Kabul University of Medical Sciences Kabul Afghanistan; ^6^ Guangdong Provincial Key Laboratory of Intelligent Food Manufacturing Foshan University Foshan China; ^7^ School of Food Science and Engineering South China University of Technology Guangzhou China

**Keywords:** 2,2‐diphenyl‐1‐picrylhydrazyl (DPPH), antioxidant potential, flaxseed polysaccharide gum (FPG), hyperlipidemia

## Abstract

Flaxseed polysaccharide gum (FPG) was extracted through the ultrasound‐assisted process using water as a solvent with a yield ranging from 8.05 ± 0.32% to 12.23 ± 0.45% by changing different extraction variables. The extracted FPG was analyzed for its functional groups and antioxidant potential. The maximum DPPH (2,2‐diphenyl‐1‐picrylhydrazyl) free radical scavenging activity (≈100%) of FPG was noted at concentrations beyond ≈10 mg·ml^−1^. The maximum inhibition percentage through ABTS (2,2′‐azino‐bis 3‐ethylbenzothiazoline‐6‐sulfonic acid) (72.4% ± 1.9%) was noted at 40 mg·ml^−1^, which was observed to be less when compared to DPPH at the same concentration. The total antioxidant potential of the FPG solution at a concentration of 10 mg·ml^−1^ was equivalent to 461 mg ascorbic acid, which tends to increase with concentration at a much lower scope. The in vivo trial suggested that the least weight gain was noted in experimental groups G_2_ and Gh_2_. A significant reduction in total cholesterol was noticed in G_1_ (−14.14%) and G_2_ (−17.72%) and in Gh_1_ (−22.02%) and Gh_2_ (−34.68%) after 60 days of the trial compared to the baseline values. The maximum reduction in total triglyceride was observed in Gh_2_ (−25.06%) and Gh_1_ (−22.01%) after 60 days of the trial. It was an increasing trend in high‐density lipoprotein cholesterol (HDL‐c) in different experimental groups G_2_ (10.51%) than G_1_ (5.35%) and Gh_2_ (48.96%) and Gh_1_ (31.11%), respectively, after 60 days of study interval. Reduction of −5.05% and − 9.45% was observed in G_1_ and G_2_, while similar results were observed in Gh_1_ and Gh_2_. Conclusively, results suggested a possible protective role of FPG against hyperlipidemia.

## Graphical abstract

On the basis of current research it was suggested that FPG have anti‐hyperlipidemic potential. Still, the mechanism of actions of FPG as anti‐hyperlipidemic and other therapeutic potential have yet to be explored.

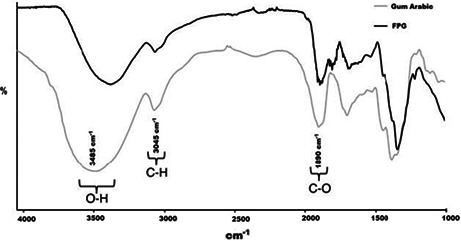

## 1 INTRODUCTION

Recent evidence has shown the beneficial role of plant polyphenols in preventing and treating various metabolic syndromes, including diabetes, cancer, obesity, stroke, hyperlipidemia, and cardiovascular complications (Liu, 2013). The lifestyle modifications and changes in dietary patterns may significantly reduce the disease burden in one's life. Plant‐derived natural compounds include lignans, polysaccharides, stilbenes, flavonoids, xanthophyll, phenolic acids, carotenes, flavonols, tannins, etc., abundantly found in various parts of plant matrices (Manzoor, Ahmad, et al., 2019; Xu et al., 2017). Among primary and secondary constituents present in plants, polysaccharides are known as key biological macromolecule that consists of homo‐ and hetero monosaccharides and uronic acid linked via glycosidic bonds. Bioactive polysaccharides are abundant in different parts of seaweed, plants, bacteria, fungi, and animals and are responsible for performing various physiological functionalities in a living organism (Ullah et al., 2019).

Extraction of bioactive components from plant‐based materials is vital and can be achieved via different techniques. The most efficient approaches include solvent extraction, hydro‐distillation, microwave‐assisted extraction, supercritical fluid extraction, and extraction through ultrasonication. Each technique has its own advantages and disadvantages (Manzoor et al., 2020; Samaram et al., 2015). Among these, ultrasonication has several advantages over other extraction techniques like low temperature and energy requirements, lower extraction time, and the quality of extracted material (Ahmed et al., 2019; Ahmed et al., 2020). Ultrasonication disrupts plant tissues via physical forces developed during the cavitation process, which results in an effective release of extractable constituents in a short time (Manzoor, Zeng, et al., 2019; Manzoor, Hussain, et al., 2021; Manzoor, Hussain, Naumovski, et al., 2022; Umego et al., 2021). Phytochemicals like polyphenols, carotenoids, aromas, and polysaccharides have been successfully extracted through ultrasonication from plant materials. Studies have shown positive effects of ultrasonication for the extraction of specific compounds, such as antioxidant compounds, polysaccharide gum, etc., using novel techniques from plant food (Chemat et al., 2020; Kumar et al., 2021; Manzoor, Hussain, Tazeddinova, et al., 2022; Manzoor, Xu, et al., 2021).

Flaxseed (*Linum usitatissimum* L.) is gaining more importance as a functional food throughout the world due to its high content of soluble and insoluble dietary fibers, linolenic acid, and lignans (secoisolariciresinol diglucoside [SDG]), which are involved in the management of cholesterol (Naik et al., 2018; Soltanian & Janghorbani, 2018). Lignans derived from flaxseed play a pivotal role in managing atherosclerosis, including potential as potent angiogenic, anti‐inflammatory, antioxidant, and anti‐apoptotic properties (Kezimana et al., 2018; Zanwar et al., 2012). Most importantly, α‐linolenic acid (ALA) present in flaxseed oil is also associated with human health advantages (Baker et al., 2016). Fibers from flaxseed have a significant role in managing serum cholesterol and serum glucose levels by reducing or otherwise delaying the absorption from the intestine (Dzuvor et al., 2018; Repin et al., 2017).

The most common method for the extraction of polysaccharide gum from different sources is aqueous extraction which is not considered time‐ and energy efficient. So, the best alternative technique is ultrasonication due to its simplicity, cost efficiency, and optimum extraction yield with fixed biological properties. This study was designed to extract polysaccharide gum from flaxseed through ultrasonication. Furthermore, the extracted polysaccharide gum was evaluated for its lipid‐lowering potential in an animal model.

## 2 MATERIAL AND METHODS

### 2.1 Procurement of raw materials

The flaxseed (*Linum usitatissimum*) cv. *Chandni* was acquired from Oilseeds Research Center, Ayub Agriculture Research Institute, Faisalabad, Pakistan. Proper cleaning of seeds was done to remove any extraneous matters. Oil extraction was carried out by Mini Oil Presser (Model 6YL‐550) at low temperature with a 2–3 kg/h capacity to obtain a partially defatted flaxseed meal. The flaxseed meal was stored until further processing at 25 ± 2°C in double zipper plastic bags of 1‐kg capacity.

### 2.2 Ultrasound‐assisted extraction of flaxseed polysaccharide gum (FPG)

Ultrasound‐assisted extraction of FPG was performed using ultrasonic (model VCX 750, Sonics & Materials, Inc.) with distilled water as the solvent by optimizing different extraction conditions (Zhong & Wang, 2010). Filtration of extracted FPG solution was carried out through a 40‐mesh screen. The filtered material was then precipitated with 95% ethanol. Centrifugation was done to separate FPG from solution, and the FPG yield was calculated as a percentage of partially defatted flaxseed meal.

### 2.3 Characterization of extracted FPG

Chemical characterization of extracted FPG was carried out by using respective methods. AACC Method No. 44‐15A was used to estimate the moisture content of FPG. Method No. 08‐01 was used to estimate ash content from the FPG sample at a temperature of 550°C using a muffle furnace (Model MF‐1/02, PCSIR, Pakistan). Method No. 30‐10 was used to determine crude fat content using the Soxhlet apparatus (Model H‐21045, Hoganas) (AACC, 2000). Crude protein was estimated by Kjeldahl's method, as mentioned in Method No. 990.03. Method No. 978.10 was used to determine the crude fiber content (AOACC, 2019). Nitrogen‐free extract (NFE) was calculated using the differentiation method mentioned below.

$$\mathrm{NFE}\left(\%\right)=100–\left(\mathrm{MC}\%+\mathrm{Ash}\%+\text{Crude}\;\mathrm{fat}\%+\text{Crude protein}\%+\text{Crude fiber}\%\right).$$

Alkaline titration was used to calculate cyanogenic compounds in the form of hydrocyanic acid (HCN) present in FPG samples according to the procedure described in AOACC (2019). To determine tannins in the FPG sample, Schanderi (1970) adopted the method.

### 2.4 Fourier transform infrared spectroscopic analysis

FPG sample was analyzed for its functional group through Fourier transform infrared (FTIR) spectroscopy with the help of Analect Instrument fx‐6160 (Irvine, CA, USA), as illustrated by Sila and coworkers (Sila et al., 2014). After mixing 1 mg of FPG sample with 100 mg potassium bromide (KBr), the transmission (%) was recorded between 1000 and 4000 cm^−1^. The similarities of the FPG sample were compared with those of the FTIR spectrum of gum arabic as standard.

### 2.5 DPPH free radical scavenging activity

The capacity of polysaccharide gum to scavenge DPPH free radicals was determined by the methods illustrated by Koubaa, Ktata, et al., 2015. Different concentrations (1–20 mg) of FPG were mixed with 0.375 ml absolute ethanol and 125 μl DPPH solution (0.02% in ethanol) after adding 0.5 ml distilled water. The resultant mixtures were mixed with vortexing and kept at room temperature in the dark environment for 60 min. In the presence of FPG, DPPH was converted to colorless or pale‐yellow color that initially had deep violet color in solution. Spectrophotometric analysis (Shimadzu UV/VIS mini 1240, Fisher Scientific) was carried out at a 517 nm wavelength, and the absorbance of the sample was measured as *A*_s_. Control reading (*A*_c_) was carried out through a standard solution without FPG under the standardized employing conditions by mixing 875 μl absolute ethanol with 125 μl DPPH solution. Similarly, a blank reading (*A*_b_) was recorded by preparing a solution by mixing different concentrations (1–20 mg) of FPG with 0.5 ml distilled water and 0.5 ml absolute ethanol without DPPH solution. The following equation was used to estimate the free radical scavenging activity (% inhibition).

$$\text{Inhibition}\left(\%\right)=\frac{A_{c}+A_{b}-A_{s}}{A_{c}}\times 100$$

### 2.6 ABTS free radical scavenging activity

ABTS (2,2′‐azino‐bis 3‐ethylbenzothiazoline‐6‐sulfonic acid) was used to determine the free radical scavenging activity of FPG solution based on the discoloration of cations (Re et al., 1999). A mixture was made with 7 μM ABTS using distilled water, and a solution of 2.45 μM potassium persulfate was added. The prepared solution was stored at normal temperature for a time duration of 16 h. The ABTS^•+^ radical cations resulted in an intense color. The mixture was diluted with distilled water, and the absorbance was measured at 734 nm. The reaction mixture was kept at room temperature for 6 min, and then 10 μl of FPG solution at different concentrations was mixed with 1 ml of ABTS^•+^ diluted solution (*A*_734 nm_ = 0.7 ± 0.02). It shows the antioxidant molecules' capacity to prevent ABTS radical oxidation. The following equation was calculated to ABTS^•+^ scavenging activity in terms of % inhibition of FPG solution.

$$\text{Inhibition}\left(\%\right)=\left(1+\frac{A}{A_{0}}\right)\times 100$$

where *A*: Solutions containing the free radical ABTS^•+^ and *A*_0_: Solutions without the free radical ABTS^•+^.

### 2.7 Total antioxidant activity

The total antioxidant activity of FPG was determined by the method used by Koubaa et al., 2015 with some modifications. Different concentrations of FPG (5, 7.5, 10, and 15 mg) were taken in tubes, and then 1 ml of reagent solution (4 mM ammonium molybdate, 28 mM sodium phosphate, and 0.6 M sulfuric acid) was added. Distilled water was used up to 1.1 ml to make the volume in each tube, and all tubes were kept in a thermostatic water bath by maintaining a higher temperature of 90°C for 1.5 h. Afterward, the tubes were cooled down to room temperature, the total antioxidant activity was measured at 695 nm, and a standard curve was used to express it as ascorbic acid equivalent (AAE). Similarly, using the above procedure, a blank reading was taken by adding 1 ml of the standard reagent with 100 μl distilled water.

### 2.8 Efficacy trials

#### 2.8.1 Experimental animals and diets

Ethical guidelines of the parent institute were followed for approval of the study design. For this research, male rats (age 7–8 weeks, body weight ranging from 160 ± 10 g) were acquired from the National Institute of Health, Islamabad, Pakistan. To normalize with conditions, the rats were kept in properly designed cages under controlled conditions of a temperature of 25 ± 2°C and relative humidity of 60 ± 5% with proper ventilation and a 12 h light–dark cycle. Standardized feed was prepared to contain 65% starch, 10% corn oil, 10% casein, 3% salt mixture, and 1% vitamins' mixture, and all groups of rats were fed with a basal diet. On the other hand, adding 10% cellulose and 1% cholesterol for hyperlipidemia is used in the standard diet. For baseline serum profile, the rats (*n* = 10) were slaughtered at the start of the study. Different biomarkers of total cholesterol, triglycerides, low‐density lipoprotein cholesterol (LDL‐c), and high‐density lipoprotein cholesterol (HDL‐c), confirmed hyperlipidemia in experimental rats. Then, normal experimental group G_0_ was given a basal diet, whereas other normal rat groups G_1_ and G_2_ were given diets comprising of FPG at 125 mg and 250 mg, respectively, up to the study trial of 60 days.

Similarly, hyperlipidemia‐induced rat group G_h0_ was given a basal diet, whereas other hyperlipidemic rat groups, G_h1_ and G_h2_, were given diets comprising FPG at 125 and 250 mg, respectively, up to the study trial of 60 days. Each of the groups mentioned above consisted of 10 experimental rats. Feed utilization by each experimental rat was estimated by subtracting the remaining spilled diet from the total quantity of diet supplied each day (Wolf, 2003). Body weight gain in experimental rats was observed after 30‐ and 60‐days' duration.

#### 2.8.2 Serum collection

Blood samples were collected on days 0, 30, and 60 through the cardiac puncture technique, and the method of Uchida et al. (2001) was used for collecting blood serum samples.

#### 2.8.3 Serum lipid profile

CHOD–PAP method was used to determine serum cholesterol levels, as illustrated by Stockbridge et al. (1989). Annoni et al. (1982) used a method to estimate the levels of total triglycerides in all serum samples by liquid triglycerides (GPO–PAP). McNamara et al. (1990) employed the method to determine the levels of low‐density lipoprotein cholesterol (LDL‐c). High‐density lipoprotein cholesterol (HDL‐c) precipitant method was employed to estimate the HDL‐c level in serum samples, as previously described by Assmann (1979).

### 2.9 Statistical analysis

Triplicate readings were carried out for all the analyses, and average values were measured to interpret the results. Duncan's multiple range (DMR) test was used to estimate the significance level among the calculated mean values. Triplicate readings were recorded for biochemical parameters, and significant differences were measured at 5% significance using statistical software Minitab version 14.1 (Steel & Torrie, 1997).

## 3 RESULTS AND DISCUSSION

### 3.1 Ultrasound‐assisted extraction of FPG

The yield of FPG by different ultrasonication runs fluctuated from 8.05 ± 0.32% to 12.23 ± 0.45%. The results concerning the yield of FPG revealed that ultrasound‐assisted extraction conditions significantly affect the gum separation and thus improve the yield of FPG. This study follows the previously conducted research for extracting FPG from partially defatted flaxseed meal under specified extraction conditions (Akhtar et al., 2019). Cui et al. (1994) optimized the extraction parameters for polysaccharide gums' extraction from seed at 85–90°C, solvent to solid ratio 13, and 6.5–7.0 extraction pH. Similarly, Maherani et al. (2007) studied the effects of independent variables such as temperature (45–100°C), solvent to solid ratio (4–24), and pH (3–7) by using response surface methodology and on the dependent variables like polysaccharide gum yield, apparent viscosity, and protein content. It was concluded from the study that two main variables, including extraction temperature and extraction pH, have a significant effect on PSG yield, whereas remaining independent factors, such as solvent to solid ratio, have a minor effect on PSG yield. Maherani et al. (2007) optimized the FPG extraction conditions as extraction temperature 85–90°C and extraction pH 6.5–7.0, keeping solvent to solid ratio 14. A study observed similar results on FPG yield carried out by Akhtar et al. (2019) using response surface methodology through sonication. So, oilseed meals are considered to be a viable source for extraction of functional food components with economic and commercial importance, and there has been much potential for extraction of functional food ingredients of commercial importance and use as a new source of functional additives in different food formulations such as to be used as hydrocolloids (Table 1).

**TABLE 1:** Chemical characterization of flaxseed polysaccharide gum (FPG) extracted through sonication

Analyzed parameter	Composition
Moisture content (%)	5.89 ± 0.37
Ash content (%)	12.06 ± 0.42
Crude protein (%)	12.94 ± 0.24
Crude fat (%)	0.78 ± 0.13
Nitrogen‐free extract (NFE) (%)	68.33 ± 0.76
Hydrocyanic acid (HCN) (mg/kg)	24.21 ± 0.64
Tannin (mg/100 g)	42.81 ± 0.15
Total dietary fibers (%)	21.78 ± 0.12
Soluble dietary fibers (%)	14.63 ± 0.11
Insoluble dietary fibers (%)	7.15 ± 0.09

*Note*: The values are mean of three replicates ± SD (*n* = 3).

### 3.2 FTIR characterization

**FIGURE 1:**
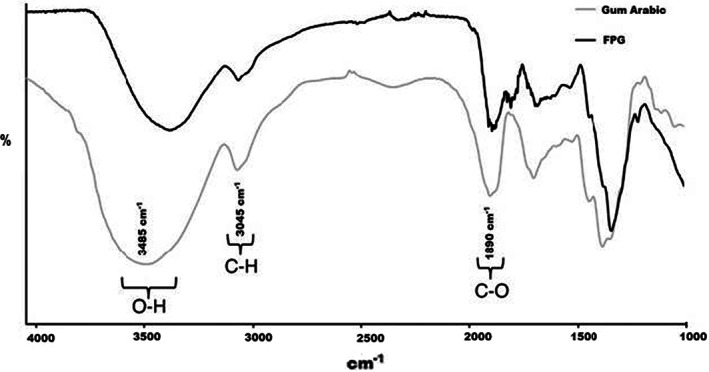
Fourier transform infrared (FTIR) spectra of flaxseed polysaccharide gum (FPG) and gum arabic (recorded between 1000 cm^−1^ and 4000 cm^−1^)

The ultrasound‐assisted extracted FPG was characterized for its functional group by FTIR (1000–4000 cm^−1^) by comparing it to the spectrum obtained from gum arabic (Figure 1). The FTIR analysis provides three distinctive differences of polysaccharides noted in samples; at 3000–3500 cm^−1^ and 1500–2000 cm^−1^. The results of the current work were in line with the studies conducted earlier by Sila et al. (2014), Wu (2009), and Yao et al. (2013). Stretching of OH groups was observed by the peak at ≈3485 cm^−1^, while the peak at 3045 cm^−1^ characterized stretching and bending vibrations of C–H (Ahmed et al., 2021; Sila et al., 2014). Furthermore, the vibrations of C–O bonds were characterized by the peak at 1890 cm^−1^. Previous studies have clearly illustrated that the pyranose present in the structure of FPG is closely linked to the incidence of the bands at 1405 cm^−1^ and 1357 cm^−1^ (Hua et al., 2014; Sila et al., 2014; Zhao et al., 2005). The results obtained from current research showed huge similarities in the functional groups of FPG and gum arabic, which depicted that FPG has an almost similar polysaccharide structure to gum arabic. Therefore, the FTIR analysis explains the types of polysaccharides present in FPG. Similar observations have previously been reported for gum extracted from different sources like red prickly pear peels (Koubaa et al., 2015); almond and pistachio processing by‐products (Sila et al., 2014), and for gum arabic and almond gum (Bouaziz et al., 2015; Bouaziz et al., 2016).

### 3.3 DPPH free radical scavenging activity

**FIGURE 2:**
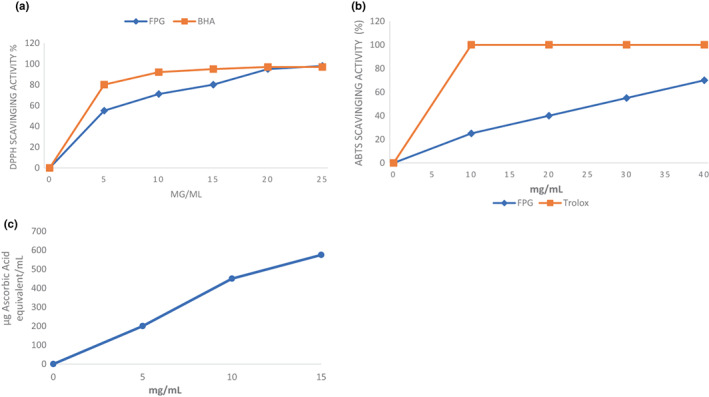
Antioxidant activities of flaxseed polysaccharide gum (FPG). (a) 2,2‐diphenyl‐1‐picrylhydrazyl (DPPH) scavenging activity, (b) 2,2′‐Azino‐bis 3‐ethylbenzothiazoline‐6‐sulfonic acid (ABTS) scavenging activity; (c) Total antioxidant capacity.

Antioxidant molecules have the potential to reduce the stable radical 1,1‐diphenyl‐2‐picrylhydrazyl (DPPH), converting deep violet color to the yellow‐colored DPPH in solution. Therefore, the DPPH test was used to determine the free radical scavenging activity of FPG (Figure 2a). The values obtained for percentage inhibition suggested that the inhibition was directly proportional to the concentration of FPG in the solution. The maximum DPPH free radical scavenging potential (≈100%) of FPG was noted at concentrations beyond ≈10 mg·ml^−1^. FPG was also analyzed for half inhibition concentration (IC_50_) value, corresponding to 4.8 mg·ml^−1^. The obtained results suggested the maximum DPPH scavenging activity was observed at the minimum IC_50_ value, which suggested less IC_50_ value of FPG than those of polysaccharides extracted from other sources like red pear peels (IC_50_ = 10.8 mg·ml^−1^); guara fruits (IC_50_ = 10.8 mg·ml^−1^) and by‐products obtained from almond juice processing (IC_50_ = 2.87 mg·ml^−1^) and was more than those of garlic straw (IC_50_ = 740 μg·ml^−1^) and by‐products from pistachio juice processing (IC_50_ = 1.61 mg·ml^−1^) (Hua et al., 2014; Kallel et al., 2015; Koubaa et al., 2015; Sila et al., 2014). FPG exhibited good scavenging activity compared with BHA, showing its capacity to react with free radicals and then convert to simpler and more stable products.

### 3.4 ABTS free radical scavenging activity

ABTS has similar scavenging potential to that of DPPH free radical. More stable compounds from free radicals have also been formed through ABTS, just like DPPH. Results for ABTS free radical scavenging activity are represented in Figure 2b, which shows that the concentration of FPG in solution has a proportional effect on the free radical scavenging activity. The maximum inhibition percentage (72.4% ± 1.9%) was noted at 40 mg·ml^−1^, which was less when compared to DPPH at the same concentration. The IC_50_ value was also noted, corresponding to 25.4 mg·ml^−1^. Though, lower values of ABTS scavenging activity were observed using Trolox as standard. Similar observations were observed by Bouaziz et al. (2016) as the scavenging ABTS radicals 75.6% at a concentration of 40 mg·ml^−1^ from FPG extracted from hulls of flaxseed. A study by Liang et al. (2017) observed ABTS radical scavenging activity at 91.2% at a concentration of 25 mg·ml^−1^, which is higher than current research.

### 3.5 Total antioxidant capacity

Total antioxidant capacity (TAC) was estimated by reducing the phosphomolybdate. At acidic pH, the green complex of phosphate/Mo (V) formed, and its absorbance was noted at 695 nm spectra. As illustrated earlier, the TAC obtained from the FPG solution was estimated as ascorbic acid equivalent (AAE)/ml (Figure 2c). The current research outcomes indicated that the concentration of FPG in solution imparts a positive impact proportional to concentration with linearity up to 10 mg·ml^−1^. At this concentration of FPG solution, the TAC was equivalent to 461 mg of ascorbic acid. The increasing trend of TAC continues with a lower slope when the concentration solution increases, representing saturation of the medium. Though the results from the current study showed lesser antioxidant potential when compared with ascorbic acid, the obtained results represent the interesting natural antioxidant potential of FPG solution. Bouaziz et al. (2016) observed a similar trend in TAC, with an increase in the concentration of FPG up to 10 mg·ml^−1^. The main components associated with the TAC of polysaccharide gums are molecular weight, solubility, structural conformation, monosaccharide composition, intermolecular hydrogen bonding, and polarity. The polysaccharide gums with lower molecular weight exhibit higher TAC. So, keeping current research and previous studies, several factors were involved in TAC polysaccharide gums (Wang et al., 2017).

### 3.6 Efficacy studies

#### 3.6.1 Effect on weight

**FIGURE 3:**
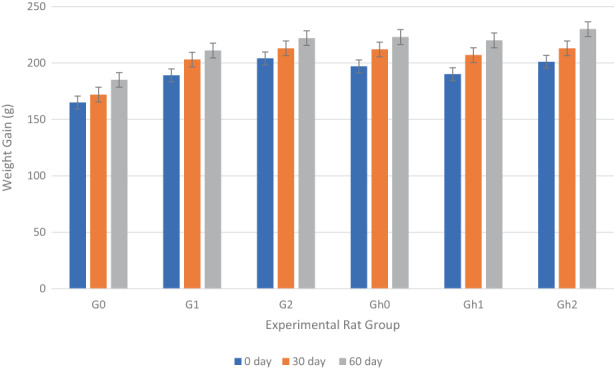
Weight gain of various groups of experimental rats during 60 days of study trial, where; G_0_: Normal group of experimental rats fed with basal diet. G_1_: Normal group of experimental rats fed with a basal diet supplemented with 125 mg flaxseed polysaccharide gum (FPG). G_2_: Normal group of experimental rats fed with a basal diet supplemented with 250 mg FPG. Gh_0_: Hyperlipidemic group of experimental rats fed with a hypercholesteremic diet. Gh_1_: Hyperlipidemic group of experimental rats fed with a hypercholesteremic diet supplemented with 125 mg FPG. Gh_2_: Hyperlipidemic group of experimental rats fed with a hypercholesteremic diet supplemented with 250 mg FPG.

In this research, a significant reduction in body weight was observed in the hyperlipidemic group of experimental rats compared to the normal group of experimental rats (Figure 3). Maximum weight gain in normal experimental rats was noted in G_0_ followed by G_1_, whereas on the other hand, in G_2,_ the lowest increase was observed after 30 days of studies. For normal experimental rats, similar observations were recorded after 60 day of trials. Almost similar observations were recorded for hyperlipidemic groups of experimental rats as the maximum weight gain was observed in Gh_0_, while the experimental group Gh_2_ showed the lowest weight gain after 30 days of study. Similarly, after 60 days of trial, the maximum gain in weight was recorded in Gh_0_ followed by Gh_1,_ and the least gain in weight was noted in Gh_2_.

The current research showed that FPG could reduce body weight in experimental rats due to its special dietary fiber ingredients. The FPG possibly improves the absorption of water, thus resulting in increased intestinal volume, constipation, and ease in defecation, ultimately resulting in weight management (Singh et al., 2011).

#### 3.6.2 Effect on total cholesterol

The experimental groups of normal and hyperlipidemic rats were observed for their serum lipid profile during the research trial after 30‐ and 60‐day time intervals (Table 2). The maximum significant reduction of −9.39% was noted in group G_2_ in normal experimental rats after 30 days of the study trial, while a significant reduction of −8.23% was observed in G_1_ of experimental rats. Furthermore, a highly significant reduction was observed in group G_2_ (−17.72%) of experimental rats after 60 days and in group G_1_ (−14.14%). Overall, a significant reduction was noted in hyperlipidemic groups of experimental rats. A significant reduction was observed in G_h1_ (−9.50%), but the reduction was lower than Gh_2_ (−17.35%) during the 30‐day study trial as compared with the control group. A significant reduction in total cholesterol level was observed in Gh_1_ (−22.02%) and Gh_2_ (−34.68%) after 60 days compared to baseline values.

**TABLE 2:** A total cholesterol level of various groups of experimental rats in 60 days of research trial

Rat group	Study duration (mean value)			Net change		% change	
	0‐day	30‐day	60‐day	0–30 day	0–60 day	0–30 day	0–60 day
G_0_	94.23 ± 1.96^Ba^	96.85 ± 2.03^Da^	97.09 ± 2.12^Da^	2.62 ± 0.11	2.86 ± 0.14	2.78	3.03
G_1_	96.03 ± 2.07^Ba^	88.12 ± 1.83^Eb^	82.45 ± 2.01^Ec^	−7.91 ± 0.17	−13.58 ± 0.91	−8.23	−14.14
G_2_	96.93 ± 2.14^Ba^	87.82 ± 1.91^Eb^	79.75 ± 1.95^Ec^	−9.11 ± 0.65	−17.18 ± 0.96	−9.39	−17.72
Gh_0_	195.36 ± 3.82^Ac^	241.31 ± 2.89^Ab^	280.11 ± 3.43^Aa^	45.95 ± 1.12	84.75 ± 1.43	23.52	43.38
Gh_1_	194.51 ± 4.01^Aa^	182.03 ± 3.98^Bb^	151.67 ± 4.16^Bc^	−18.48 ± 0.87	−42.84 ± 1.03	−9.50	−22.02
Gh_2_	196.81 ± 3.78^Aa^	162.65 ± 3.97^Cb^	128.54 ± 4.03^Cc^	−34.16 ± 1.24	−68.27 ± 1.85	−17.35	−34.68

*Note*: Mean ± Standard deviation. Where G_0_: Normal group of experimental rats fed with basal diet. G_1_: Normal group of experimental rats fed with a basal diet supplemented with 125 mg flaxseed polysaccharide gum (FPG). G_2_: Normal group of experimental rats fed with a basal diet supplemented with 250 mg FPG. Gh_0_: Hyperlipidemic group of experimental rats fed with a hypercholesteremic diet. Gh_1_: Hyperlipidemic group of experimental rats fed with a hypercholesteremic diet supplemented with 125 mg FPG. Gh_2_: Hyperlipidemic group of experimental rats fed with a hypercholesteremic diet supplemented with 250 mg FPG. Means showing different uppercase letters represent significant differences (*p* < .05) within the control or hyperlipidemic experimental groups. Whereas means showing different lowercase letters represent significant differences (*p* < .05) across a 0‐to‐60‐day trial.

One of the most important reasons for lowering the total cholesterol level is the comparatively high viscosity of FPG. The comparatively high viscosity of FPG resulted in delaying the digestion time and thus the absorption of food by increasing the weight and viscosity of chyme in the stomach of rats; ultimately, gastric emptying is delayed, thus prolonging the retention time of chyme in the different parts of the intestine. All these processes slowed down food digestion in the digestive tract. All these phenomena and mechanisms resulted in a reduction in total cholesterol within the blood serum (Chen et al., 2020; Fabek & Goff, 2015). Another possible reason for a reduction in total cholesterol level is the emulsifying properties of FPG, as FPG may form an emulsion layer with fat droplets causing depletion in flocculation and resulting in a reduction of the bioavailability of fat and cholesterol (Minekus et al., 2005; Ohashi et al., 2012). One possible reason for lowering the total cholesterol level of FPG is its prebiotic effect, as microorganisms have also been found to have a cholesterol‐lowering effect. Microorganisms ferment FPG in the intestine to produce short‐chain fatty acids like propionate, which ultimately affect the synthesis of Acetyl‐coenzyme A (CoA) synthetase, which results in inhibition of the synthesis of cholesterol and fatty acids within the body (Wang et al., 2021).

#### 3.6.3 Effect on triglyceride

A significant reduction in total triglyceride was noted in G_1_ (−6.39%) and G_2_ (−11.66%) of normal experimental rats compared to G_0_ after 30 days (Table 3). After the 60 days of the experimental trial, a significant reduction was noted in the G_1_ and G_2_ of normal experimental rats. On the other hand, a reduction in total triglyceride was observed in Gh_1_ (−12.67%) and Gh_2_ (−13.74%) of hyperlipidemic experimental rats compared with baseline data during 30 days of study. The maximum reduction of −25.06% and −22.01% was observed in Gh_2_ and Gh_1,_ respectively, after 60 days of trial. The current research outcomes highlighted the importance of FPG in managing triglyceride levels among all experimental groups (Table 3).

**TABLE 3:** Triglyceride level of various groups of experimental rats in 60 days of research trial

Rat group	Study duration (mean value)			Net change		% change	
	0‐day	30‐day	60‐day	0–30 day	0–60 day	0–30 day	0–60 day
G_0_	102.54 ± 3.04^Da^	103.78 ± 2.93^Da^	105.13 ± 2.89^Da^	1.24 ± 0.14	2.59 ± 0.62	1.20	2.52
G_1_	119.25 ± 3.16^Ca^	111.62 ± 2.96^Cb^	104.33 ± 2.73^Dc^	−7.63 ± 0.88	−14.92 ± 1.14	−6.39	−12.51
G_2_	94.72 ± 2.23^Ea^	83.67 ± 2.07^Eb^	73.42 ± 2.48^Ec^	−11.05 ± 1.03	−21.30 ± 1.41	−11.66	−22.48
Gh_0_	146.31 ± 3.42^Bc^	163.58 ± 4.02^Ab^	195.45 ± 4.15^Aa^	17.27 ± 1.17	49.14 ± 2.01	11.80	33.58
Gh_1_	162.71 ± 3.56^Aa^	142.08 ± 3.63^Bb^	126.91 ± 3.24^Bc^	−20.63 ± 1.24	−35.8 ± 1.76	−12.67	−22.01
Gh_2_	161.81 ± 3.48^Aa^	139.57 ± 2.98^Bb^	121.25 ± 3.18^Cc^	−22.24 ± 1.37	−40.56 ± 1.83	−13.74	−25.06

*Note*: Mean ± Standard deviation. Where G_0_: Normal group of experimental rats fed with basal diet. G_1_: Normal group of experimental rats fed with a basal diet supplemented with 125 mg flaxseed polysaccharide gum (FPG). G_2_: Normal group of experimental rats fed with a basal diet supplemented with 250 mg FPG. Gh_0_: Hyperlipidemic group of experimental rats fed with a hypercholesteremic diet. Gh_1_: Hyperlipidemic group of experimental rats fed with a hypercholesteremic diet supplemented with 125 mg FPG. Gh_2_: Hyperlipidemic group of experimental rats fed with a hypercholesteremic diet supplemented with 250 mg FPG. Means showing different uppercase letters represent significant differences (*p* < .05) within the control or hyperlipidemic experimental groups. Whereas means showing different lowercase letters represent significant differences (*p* < .05) across a 0‐to‐60‐day trial.

To better understand the effects of FPG on serum triglyceride levels, the studies above concluded that the viscous fiber reduced the postprandial triglyceride level in an animal model (Reimer et al., 2011). Studies have shown no significant association between FPG consumption and its effect on triglycerides. The regulatory mechanism is performed through the triglyceride homeostasis mechanism by lipid droplets (LDs) and smooth endoplasmic reticulum (sER) in the periphery of hepatocytes (Rai et al., 2017). A similar observation has also been noted by Kristensen et al. (2012), who also found a gradually decreasing trend in triglyceride levels in experimental subjects.

#### 3.6.4 Effect on high‐density lipoprotein cholesterol (HDL‐c) level

Significant differences were noted in HDL‐c levels in experimental groups among all categories of hyperlipidemic rats. A nonsignificant difference was observed in the normal group of experimental rats after 30 days compared with baseline data. The increasing trend was observed in G_2_ (10.51%) than in G_1_ (5.35%) compared to control group G_0_ after 60 days of the study trial. On the other hand, for hyperlipidemic rats maximum increase was observed in Gh_2_ (26.19%) than in Gh_1_ (20.92%) after 30 days of the study trial. The increasing trend was also observed in Gh_2_ (48.96%) and Gh_1_ (31.11%), respectively, after 60 days of the study trial (Table 4).

**TABLE 4:** High‐density lipoprotein cholesterol (HDL‐c) level of various groups of experimental rats in 60 days of research trial

Rat group	Study duration (mean value)			Net change		% change	
	0‐day	30‐day	60‐day	0–30 day	0–60 day	0–30 day	0–60 day
G_0_	50.23 ± 1.13^Aa^	52.47 ± 1.32^Aa^	51.31 ± 1.05^Aa^	2.24 ± 0.34	1.08 ± 0.08	4.45	2.15
G_1_	51.17 ± 1.09^Aa^	52.62 ± 1.41^Aa^	53.91 ± 1.13^Aa^	1.45 ± 0.12	2.74 ± 0.42	2.83	5.35
G_2_	49.74 ± 1.21^Ab^	52.11 ± 1.39^Aa^	54.97 ± 1.23^Aa^	2.37 ± 0.37	5.23 ± 0.54	4.76	10.51
Gh_0_	29.78 ± 0.96^Ba^	28.14 ± 1.02^Ca^	26.09 ± 0.91^Db^	−1.64 ± 0.21	−3.69 ± 0.47	−5.50	−12.39
Gh_1_	29.92 ± 0.92^Bc^	36.18 ± 1.14^Bb^	39.23 ± 1.15^Ca^	6.26 ± 0.67	9.31 ± 0.86	20.92	31.11
Gh_2_	30.04 ± 1.01^Bc^	37.91 ± 1.20^Bb^	44.75 ± 1.19^Ba^	7.87 ± 0.73	14.71 ± 0.94	26.19	48.96

*Note*: Mean ± Standard deviation. Where G_0_: Normal group of experimental rats fed with basal diet. G_1_: Normal group of experimental rats fed with a basal diet supplemented with 125 mg flaxseed polysaccharide gum (FPG). G_2_: Normal group of experimental rats fed with a basal diet supplemented with 250 mg FPG. Gh_0_: Hyperlipidemic group of experimental rats fed with a hypercholesteremic diet. Gh_1_: Hyperlipidemic group of experimental rats fed with a hypercholesteremic diet supplemented with 125 mg FPG. Gh_2_: Hyperlipidemic group of experimental rats fed with a hypercholesteremic diet supplemented with 250 mg FPG. Means showing different uppercase letters represent significant differences (*p* < .05) within the control or hyperlipidemic experimental groups. Whereas means showing different lowercase letters represent significant differences (*p* < .05) across a 0‐to‐60‐day trial.

LDL‐c, the main indicator of cardiovascular diseases, is converted through oxidation, glycosylation, and carbamylation (Alique et al., 2015). Studies have shown that the increase in LDL‐c levels is mainly responsible for atherosclerotic cardiovascular disease (Gao et al., 2017). Higher levels of LDL‐c have also been found to accelerate the risk factors of carotid atherosclerosis before the clinical event (Gao et al., 2018).

#### 3.6.5 Effect on low‐density lipoprotein cholesterol (LDL‐c) level

There was an increase in the LDL‐c level after feeding with a basal diet and a hypercholesteremic diet (Table 5). On the other hand, after giving a diet supplemented with FPG, a significant reduction in LDL‐c levels was observed in experimental rats. Reduction of −2.25% to −4.36% was noted in G_1_ and G_2_ of normal experimental rats during 30 days, and almost similar results were also noted, even after 60 days of experimental trial (−5.05% to −9.45%) compared with baseline data. On the other hand, the reduction in LDL‐c level after 30–60 days of an experimental trial in hyperlipidemic rats was higher in Gh_1_ and Gh_2_ (−16.44% and‐18.82%) after 30 days of the study trial than that of normal groups. After 60 days of the experimental trial, the LDL‐c level was reduced to normal, as shown in Table 5.

**TABLE 5:** Low‐density lipoprotein cholesterol (LDL‐c) level of various groups of experimental rats in 60 days of research trial

Rat group	Study duration (mean value)			Net change		% change	
	0‐day	30‐day	60‐day	0–30 day	0–60 day	0–30 day	0–60 day
G_0_	133.45 ± 2.16^Ba^	134.03 ± 1.94^Ba^	133.94 ± 1.83^Ba^	0.58 ± 0.07	0.49 ± 0.08	0.43	0.367
G_1_	140.94 ± 2.54^Aa^	137.76 ± 2.02 ^Ba^	133.82 ± 1.91^Bb^	−3.18 ± 0.19	−7.12 ± 0.31	−2.25	−5.05
G_2_	137.89 ± 2.09^Ba^	131.87 ± 1.81^Cb^	124.85 ± 1.62^Cc^	−6.02 ± 0.23	−13.04 ± 0.47	−4.36	−9.45
Gh_0_	139.97 ± 2.10^Ac^	163.45 ± 2.47^Ab^	185.67 ± 2.42^Ac^	23.48 ± 0.41	45.7 ± 0.78	16.77	32.64
Gh_1_	142.81 ± 2.33^Aa^	119.32 ± 1.01^Db^	94.29 ± 1.05^Dc^	−23.49 ± 0.45	−48.52 ± 0.81	−16.44	−33.97
Gh_2_	142.22 ± 2.41^Aa^	115.45 ± 1.03^Db^	79.35 ± 0.99^Ec^	−26.77 ± 0.47	−62.87 ± 0.92	−18.82	−44.20

*Note*: Mean ± Standard deviation. Where G_0_: Normal group of experimental rats fed with basal diet. G_1_: Normal group of experimental rats fed with a basal diet supplemented with 125 mg flaxseed polysaccharide gum (FPG). G_2_: Normal group of experimental rats fed with a basal diet supplemented with 250 mg FPG. Gh_0_: Hyperlipidemic group of experimental rats fed with a hypercholesteremic diet. Gh_1_: Hyperlipidemic group of experimental rats fed with a hypercholesteremic diet supplemented with 125 mg FPG. Gh_2_: Hyperlipidemic group of experimental rats fed with a hypercholesteremic diet supplemented with 250 mg FPG. Means showing different uppercase letters represent significant differences (*p* < .05) within the control or hyperlipidemic experimental groups. Whereas means showing different lowercase letters represent significant differences (*p* < .05) across a 0‐to‐60‐day trial.

The increase in the concentration of HDL‐c was due to the increased fiber intake in FPG. The current research results were in line with the findings of other studies (Dall'Alba et al., 2013; Mohamed et al., 2015).

## 4 CONCLUSIONS

This research estimated the physiochemical properties and antioxidant potential of extracted FPG. Results of the following research showed great antioxidant potential compared to commercially available standards. Similar functional groups have been observed in FPG compared to gum arabic, suggesting the similarities in polysaccharides' structure. The results of the current study suggested the potential of FPG as a coproduct to replace other commercially available gums, just like gum arabic. The potential therapeutic of FPG against hyperlipidemia was evaluated in this study. It is quite evident from current research that FPG supplementation improved biomarkers like total cholesterol, triglyceride, LDL‐c, and HDL‐c. Therefore, based on current research, it was suggested that FPG has antihyperlipidemic potential. Still, the mechanisms of action of FPG as antihyperlipidemic and other therapeutic potential have yet to be explored.

## Data Availability

The dataset supporting the conclusions of this article is included within the article.
